# Genotypic and phenotypic diversity of *Ralstonia pickettii *and *Ralstonia insidiosa *isolates from clinical and environmental sources including High-purity Water. Diversity in *Ralstonia pickettii*

**DOI:** 10.1186/1471-2180-11-194

**Published:** 2011-08-30

**Authors:** Michael P Ryan, J Tony Pembroke, Catherine C Adley

**Affiliations:** 1 Microbiology Laboratory, Department of Chemical and Environmental Sciences, University of Limerick, Limerick, Ireland; 2 Molecular and Structural Biochemistry Laboratory, Department of Chemical and Environmental Sciences, University of Limerick, Limerick, Ireland

**Keywords:** *Ralstonia pickettii*, random amplified polymorphic DNA, PCR, genotyping, High-Purity Water

## Abstract

**Background:**

*Ralstonia pickettii *is a nosocomial infectious agent and a significant industrial contaminant. It has been found in many different environments including clinical situations, soil and industrial High Purity Water. This study compares the phenotypic and genotypic diversity of a selection of strains of *Ralstonia *collected from a variety of sources.

**Results:**

*Ralstonia *isolates (fifty-nine) from clinical, industrial and environmental origins were compared genotypically using i) Species-specific-PCR, ii) PCR and sequencing of the 16*S-*23*S *rRNA Interspatial region (ISR) iii) the *fliC *gene genes, iv) RAPD and BOX-PCR and v) phenotypically using biochemical testing. The species specific-PCR identified fifteen out of fifty-nine designated *R. pickettii *isolates as actually being the closely related species *R. insidiosa*. PCR-ribotyping of the 16*S-*23*S *rRNA ISR indicated few major differences between the isolates. Analysis of all isolates demonstrated different banding patterns for both the RAPD and BOX primers however these were found not to vary significantly.

**Conclusions:**

*R. pickettii *species isolated from wide geographic and environmental sources appear to be reasonably homogenous based on genotypic and phenotypic characteristics. *R. insidiosa *can at present only be distinguished from *R. pickettii *using species specific PCR. *R. pickettii *and *R. insidiosa *isolates do not differ significantly phenotypically or genotypically based on environmental or geographical origin.

## Background

*Ralstonia pickettii*, previously called *Pseudomonas pickettii *and *Burkholderia pickettii *[1], is ubiquitous in the environment. It has been recovered from a number of water sources and from a wide range of clinical environments [2-5]. *R. pickettii *has also become recognised in the last decade as a nosocomial pathogen associated particularly with individuals who are debilitated or immunosuppressed [6-8]. These outbreaks have been reported mainly in association with contamination of hospital supplies [9-14] and with contaminated chlorhexidine skin cleansing solutions [15,16]. The emergence of new opportunistic pathogenic microorganisms has been linked to a multiresistance phenotype that makes them refractory to the antibiotics commonly used in clinical practice [17]. The majority of clinical isolates of *R. pickettii *are characterized by their multiresistance to common antibiotics [17].

The emergence of *R. pickettii *in High-Purity Water (HPW) systems used in the biopharmaceutical industry necessitates revisiting this organism. *R. pickettii *has been identified in biofilm formation in industrial plastic water piping [18] and has been isolated from industrial high-purity water [2,19]; laboratory based high-purity water systems [3]; in the Space Shuttle water system [20] and from the Mars Odyssey probe encapsulation facility [21]. It has been shown to produce homoserine lactones [2], the putative cell-cell signalling molecules in biofilm development [22] and has the ability to survive in low nutrient (oligotrophic) conditions [23]. In addition, *R. pickettii *has been shown to have a wide range of biodegradative abilities that could potentially be used for commercial applications and that may assist in survival and adaption to low nutrient environments [8]. Integrating Conjugative Elements-like elements have been discovered in several isolates of this bacterium [24] indicating a degree of plasticity in their genomes.

Molecular typing methods such as restriction fragment length polymorphism by pulsed-field gel electrophoresis (PFGE) [14,25], random amplified polymorphic DNA (RAPD) analysis by arbitrarily primed PCR [16] and ribotyping [26] have been developed for *Ralstonia *sp. and have been used to detect relationships between clinical isolates in epidemiological studies. Despite the acknowledged importance of *R. pickettii *as a nosocomial pathogen, little is known regarding its epidemiology. Studies carried out with limited numbers of bacterial isolates indicated the bacterium appears to have limited diversity [25-27].

Evidence suggests that *R. pickettii *finds its way into clinical environments through contaminated water supplies [5]. To test this and to determine the level of relatedness between isolates of this bacteria from different environments a comprehensive study of the relatedness of fifty-nine isolates of *R. pickettii *and *R. insidiosa *(including soil, water and clinical isolates) using various phenotypic (metabolic activity) and genotypic (flagellin and Interspatial regions typing, BOX-PCR, and RAPD) fingerprinting methods was carried out.

## Methods

### Bacterial isolates and growth conditions

The fifty-nine isolates used in this study are presented in Table 1. All the isolates were stored at -20°C in Nutrient Broth (Difco) with 50% glycerol. Isolates were grown aerobically on Nutrient Agar (Difco) and incubated overnight at 30°C.

**Table 1:** *Ralstonia *Isolates used in this work

Strain	Source
*R. pickettii* JCM5969, NCTC11149, DSM6297, CIP73.23 CCUG3318, CCM2846, CCUG18841	Culture Collection

*R. pickettii* ULC193, ULC194, ULC244, ULC277, ULC297, ULC298, ULC421	Microbiology laboratory of Limerick Regional Hospital (Cystic Fibrosis Patients)

*R. pickettii* ULI788, ULI790, ULI791, ULI796, ULI800, ULI801, ULI804, ULI806, ULI807, ULI818, ULI159, ULI162, ULI165, ULI167, ULI169, ULI171, ULI174, ULI181, ULI187, ULI188, ULI193	Isolated from various Industrial Purified water systems (Ireland)

*R. pickettii* ULM001, ULM002, ULM003, ULM004, ULM005, ULM006	Isolated from various Millipore Purified water systems (France)

*R. pickettii* ULM007, ULM010, ULM011	Isolated from various Millipore Laboratory Purified water systems (Ireland)

*R*. *insidiosa* ATCC4199, LMG21421	Culture Collection

*R*. *insidiosa* ULI821, ULI797, ULI785, ULI181, ULI794, ULI185, ULI166, ULI819, ULI784, ULI163, ULI795	Isolated from various Industrial Purified water systems (Ireland)

*R*. *insidiosa* ULM008, ULM009	Isolated from various Millipore Laboratory Purified water systems (Ireland)

### Phenotypic analysis

Oxidase and catalase tests were performed with Oxidase sticks (Oxoid, Basingstoke, UK) and 3% hydrogen peroxide, respectively. A number of classical phenotypic tests were performed that included BioMérieux API 20NE system (BioMérieux UK Limited, Hampshire, UK) and the Remel RapID NF Plus commercial system (Remel, Kansas, USA). A Vitek card; the Non-Fermenter Identification Card (NFC) (BioMérieux), was also used. All of the above tests were carried out as per manufacturer's instructions. Phenotypic relatedness among different isolates of *R. pickettii *was determined using the API 20NE profiles. Phenotypic characters were scored as discrete variables [0 or 1]; 0, when the character was negative or missing; 1, when character was positive or present). Isolates with the same pattern were grouped into Biotypes numbering 1 to 35. The unweighted pair group method [28] was used for cluster analysis using the MultiVariate Statistical Package (MVSP) software program ver. 3.13 by means of the Jaccard coefficient [29]. The discriminatory power of the biotyping for typing *R. pickettii *isolates was evaluated by using the discrimination index as described by Hunter and Gaston, as given by the equation: D = 1 - [1/N (N - 1)] ∑nj (nj - 1), where D is the numerical index of discrimination, N is the total number of isolates, and nj is the number of isolates pertaining to the j^th ^type [30].

### Genotypic analysis

DNA for all PCR experiments was prepared as described previously [31].

### Species-specific *PCR and *amplification 16S-23S rRNA ISR and *fliC *gene

The species-specific PCR primers (Rp-F1, Rp-R1 and R38R1) used in this study were designed by Coenye *et al.*, detailed in Table 2[32,33]. The 16*S*F and 23*S*R primers were used to amplify the Interspacial Region (ISR) [34] and the Ral_*fliC *primers (Ral_*fliC*F and Ral_*fliC*R) were used to amplify the *fliC *gene (Table 2), [35]. The PCR assays were performed in 25 μL reaction mixtures, containing 100 ng of template genomic DNA, 1U *Taq *polymerase, 250 mM (each) deoxynucleotide triphosphate, 1.5 mM MgCl_2_, 10x PCR buffer (Bioline), and 20 pmol of oligonucleotide primer (MWG Biotech, Ebersberg, Germany) Rp-F1 and 10 pmol of oligonucleotide primers Rp-R1 and R38R1 for the species-specific PCR and 20 pmol each of the primers for the ISR and *fliC *regions (Table 2). After initial denaturation for 2 min at 94°C, 30 amplification cycles were completed, each consisting of 1 min at 94°C, 1 min at 55°C, and 1 min 30 secs at 72°C. A final extension of 10 min at 72°C was then applied. The PCR products were analysed by electrophoresis in a 1.5% agarose gel (Agarose MP, Roche Diagnostics) for 1 hour (100 V) with ethidium bromide staining in the TBE buffer and photographed under the UV light (UV Products Gel Documentation System Imagestore, Ultra Violet Products, Cambridge). A 200-10000bp DNA ladder (Bioline) was included on all gels to allow standardization and sizing. Following amplification of the ISR and *fliC *region from test isolates PCR product was purified using the NucleoSpin Extract II kit (Macherey-Nagel, Düren, Germany) according to the manufacturer's instructions and the amplicons sequenced (MWG Comfort Read service).

**Table 2:** Oligonucleotides used in this study

Primer	Oligonucleotide Sequence 5'-3'	Target^a^	Product size	Reference
Rp-F1	ATGATCTAGCTTGCTAGATTGAT	16S rRNA gene	210 bp	[32]
Rp-R1	ACTGATCGTCGCCTTGGTG	16S rRNA gene		[32]
R38R1	CACACCTAATATTAGTAAGTGCG	16S rRNA gene	403 bp	[33]
16SF	TTGTACACACCGCCCGTCA	16S-23S Spacer Region	860 bp	[34]
23SR	GGTACCTTAGATGTTTCAGTTC	16S-23S Spacer Region		[34]
Ral_*fliC*F	CCTCAGCCTCAATACCAACATC	*fliC *gene	725 bp	[35]
Ral_*fliC*R	CATGTTCGACGTTTCMGAWGC	*fliC *gene		[35]
M13	TTATGTAAAACGACGGCCAGT	RAPD Primer	N\A	[36]
P3	AGACGTCCAC	RAPD Primer	N\A	[37]
P15	AATGGCGCAG	RAPD Primer	N\A	[37]
OPA03U	AGTCAGCCAC	RAPD Primer	N\A	[38]
BOX-A1R	CTACGGCAAGGCGACGCTGACG	BOX Primer	N\A	[39]

### RAPD and BOX-PCR analysis

For RAPD-PCR each 25 μl PCR sample contained 2.5 μL of 10× buffer, 2 mM of MgCl_2_, 40 pmol of primer, 200 mM of each of four dNTPs, 200 ng of template genomic DNA and 1 U of *Taq *polymerase. The PCR reactions were carried out as follows: an initial denaturation at 94°C for 5 min followed by 40 cycles of denaturation at 94°C for 1 min, annealing at 36°C for 1 min and extension at 72°C for 1 min 30 secs. Four different primers were used: M13, P3, P15 and OPA03U are listed in Table 2[36-38].

BOX-PCR typing was carried out with the BOX-A1R primer (Table 2) [39]. 200 ng of template genomic was mixed with 2 U of *Taq *polymerase, 200 mM of each of four dNTPs, 2.5 μl of dimethyl sulfoxide (DMSO), 0.8 μl of bovine serum albumin (10 mg ml^-1^) (Promega), 5 μl of 5× Gitschier buffer and 10 pmol of primer in a final volume of 25 μl. After initial denaturation for 2 min at 95°C, 35 amplification cycles were completed, each consisting of 40 secs at 94°C, 1 min at 50°C, and 8 mins at 65°C. A final extension of 8 mins at 65°C was applied.

Amplified products for both procedures were analysed by electrophoresis in a 2% agarose gel containing ethidium bromide at 60 V for 4 hrs and were visualised by UV transillumination. The repeatability of the RAPD and BOX-PCR protocols were tested by studying the isolates in three independent runs.

### DNA analysis

The ISR and *fliC *gene sequences obtained were compared with sequences in the GenBank database using the Basic Local Alignment Search Tool (BLAST) [40] and aligned using the ClustalW program [41]. Phylogenetic and molecular evolutionary analyses were conducted using genetic distance based neighbour joining algorithms [42] within MEGA version 3.1 http://www.megasoftware.net, [43]. The analysis of the RAPD and BOX gels was performed using BioNumerics software (version 5.1 Applied Maths, Kortrijk, Belgium), based on the Pearson correlation coefficient, and clustering by the unweighted pair group method with arithmetic means (UPGMA method) [44]. The isolates that clustered at a cut-off level of more than 80% similarity were grouped together; these were considered clonally related and classified into the same group. The discriminatory power of the BOX and RAPD-PCR for typing *R. pickettii *isolates was evaluated by using the discrimination index as described by Hunter and Gaston [30].

### Accession numbers

DNA sequences were deposited in the EMBL database with accession numbers for sequences from the 16*S*-23*S *spacer region are as follows: AM501933-AM501952 and for the *FliC *genes: FN869041-FN869057.

## Results

### Species-specific PCR

To confirm that the isolates were in fact *R. pickettii *a species-specific PCR reaction was carried out using the primers described (Table 2). The results of the experimental analysis of fifty-nine isolates from our study, which include industrial, clinical, laboratory purified water and seven purchased strains are presented in Table 3. Eleven of the industrial high purity water isolates (ULI821, ULI797, ULI785, ULI181, ULI794, ULI185, ULI166, ULI819, ULI784, ULI163, ULI795), two laboratory Millipore water isolates (ULM008, ULM009) and one purchased strain (ATCC42129) were identified as *R. insidiosa *through use of the species-specific primers (Table 2). The multiplex PCR gave a 403 bp band and a 210 bp band for *R. insidiosa *and only the 210 bp band for *R. pickettii *(data not shown).

**Table 3:** Characterization of Isolates of *Ralstonia *sp. using phenotypic assays and whole genome typing

Strain	API 20 NE		RapID NF Plus	Vitek (NFC)	RAPD				BOX
	**Biotype**	**% ID^A^**	**% ID^A^**	**% ID^A^**	**M13**	**OPA3OU**	**P3**	**P15**	**BOX-A1R**

***Ralstonia pickettii***									

JCM5969	B1	99.00	99.94	99.00	A	e	VIII	13	F
NCTC11149	B4	95.10	99.94	99.00	D	a	IX	13	F
DSM 6297	B4	95.10	99.94	99.00	D	e	XX	13	F
CCUG3318	B7	91.10	99.94	99.00	D	a	XIX	13	F
CIP73.23	B7	91.10	99.94	99.00	D	n	XX	13	F
CCUG18841	B30	00.00	99.71	99.00	L	k	VI	13	L
CCM2846	B30	00.00	99.71	97.00	L	k	VI	13	L

ULI 187	B3	97.70	98.34	99.00	I	e	VII	13	G
ULI 188	B4	95.10	99.99	99.00	M	k	VII	13	G
ULI 798	B5	95.10	99.99	99.00	K	k	VII	13	H
ULI 807	B10	84.10	99.99	99.00	K	k	XIX	13	F
ULI 171	B10	84.10	99.99	99.00	I	c	VI	13	G
ULI 788	B11	80.40	99.94	99.00	J	f	XIV	13	J
ULI 800	B11	80.40	99.99	99.00	I	e	XXIII	13	A
ULI 169	B11	80.40	99.99	99.00	K	k	VI	13	A
ULI 165	B14	67.90	99.99	99.00	N	e	XXIV	13	D
ULI 174	B14	67.90	98.34	99.00	A	e	XIX	13	A
ULI 193	B15	61.70	98.38	99.00	A	e	X	6	A
ULI 796	B16	60.00	98.34	99.00	H	e	X	6	A
ULI 801	B17	56.90	99.99	99.00	A	a	X	6	A
ULI 791	B17	56.90	99.99	99.00	B	j	XI	19	A
ULI 790	B20	44.80	98.34	99.00	H	m	X	10	B
ULI 818	B21	39.50	99.94	99.00	H	k	X	9	B
ULI 804	B23	24.50	98.34	99.00	B	a	XI	19	B
ULI 159	B29	00.00	99.94	99.00	F	c	X	8	B
ULI 806	B34	00.00	99.99	99.00	A	a	X	7	A
ULI 167	B33	00.00	99.94	99.00	H	k	X	9	A
ULI 162	B30	00.00	99.99	99.00	A	e	X	6	C

ULC 298	B8	90.10	99.99	99.00	A	b	X	5	K
ULC 297	B13	70.03	99.94	99.00	A	e	X	2	K
ULC 277	B15	61.70	99.99	99.00	A	b	X	1	K
ULC 244	B18	56.70	99.94	99.00	A	e	X	3	L
ULC 193	B18	56.70	98.34	99.00	A	a	X	4	K
ULC 194	B18	56.70	99.99	99.00	A	a	X	3	L
ULC 421	B21	28.50	99.99	99.00	A	a	XVI	15	P
ULM 001	B4	95.10	99.99	99.00	P	h	III	14	R
ULM 002	B4	95.10	99.99	99.00	T	h	XVI	13	Q
ULM 003	B9	88.60	99.28	99.00	R	h	XVI	13	
ULM 004	B7	91.10	99.99	99.00	S	h	XVIII	13	Q
ULM 005	B4	95.10	00.00	99.00	A	e	XVII	13	O
ULM 006	B4	95.10	99.28	99.00	Q	h	XVII	13	M
ULM 007	B4	95.10	99.99	99.00	R	h	XVI	13	M
ULM 010	B2	99.40	99.99	99.00	A	g	XVI	13	M
ULM 011	B2	99.40	99.99	99.00	A	g	XXII	13	M

***Ralstonia insidiosa***									

LMG21421	B15	61.70	99.94	99.00	E	d	XVII	13	H
ATCC49129	B6	92.40	99.99	99.00	B	b	III	14	H
ULI 821	B10	84.10	99.94	99.00	E	d	XV	18	E
ULI 797	B10	84.10	98.34	99.00	O	e	XXV	13	E
ULI 785	B19	53.10	99.99	99.00	H	l	XXI	13	B
ULI 181	B21	39.50	99.99	99.00	B	f	II	14	B
ULI 794	B24	06.40	34.18	99.00	G	f	II	14	B
ULI 185	B25	05.70	98.34	99.00	U	o	IV	12	B
ULI 166	B32	00.00	99.94	99.00	B	f	I	17	B
ULI 819	B26	00.00	99.99	99.00	C	i	V	21	B
ULI 784	B27	00.00	99.99	99.00	H	e	V	17	A
ULI 163	B28	00.00	98.34	99.00	B	j	VI	11	D
ULI 795	B35	00.00	98.34	99.00	B	f	I	20	A
ULM 008	B12	80.20	99.99	99.00	E	e	XII	16	M
ULM 009	B12	80.20	99.99	99.00	E	d	XII	16	M

^A ^% ID *Ralstonia pickettii*

### Phenotypic characterisation and identification

All isolates were Gram-negative non-fermentative rods and both oxidase and catalase positive. Fifty-nine isolates (eight from culture collections, seven clinical, eleven laboratory purified water and thirty-two industrial isolates and the *R. insidiosa *type strain LMG21421) were identified initially as *R. pickettii *(Table 3). These results were confirmed using the Vitek NFC with all isolates being identified as *R. pickettii*. The Vitek NFC identification rate ranged from 97.0 to 99.0 with two patterns being detected (Table 3). The API 20NE identification rate ranged from 0.00 to 99.4%, with thirty-five different patterns being detected. Most of the purchased culture collection isolates were identified as *R. pickettii *(except the soil isolates CCUG18841 and CCM2846) with cut-off points higher then 60%, six of the clinical isolates were identified as *R. pickettii *with cut-off points higher then 50%, while one was identified as *Pseudomonas aeruginosa *(Table 3). All 11 laboratory purified water isolates were identified as *R. pickettii *with cut-off points higher then 80%, and seventeen of the thirty-two industrial isolates were identified as *R. pickettii *species with cut-off points higher then 50%, the rest of the industrial isolates were all identified as non-*R. pickettii *species. The RapID NF Plus identification rate ranged from 0.00 to 99.9%, with five different patterns being detected. Fifty-seven isolates were identified as *R. pickettii*, with results of over 98%. The other two were identified as *Moraxella *sp (Table 3).

The *R. insidiosa *Type strain LMG21421 was identified as *R. pickettii *61.70% ('Low Discrimination' 0050577) with the API 20NE, as *R. pickettii *99.94% ('Implicit' 400414) with the RapID NF Plus and as *R. pickettii *99% on the Vitek Junior system with the NFC (Table 3).

**Figure 1:**
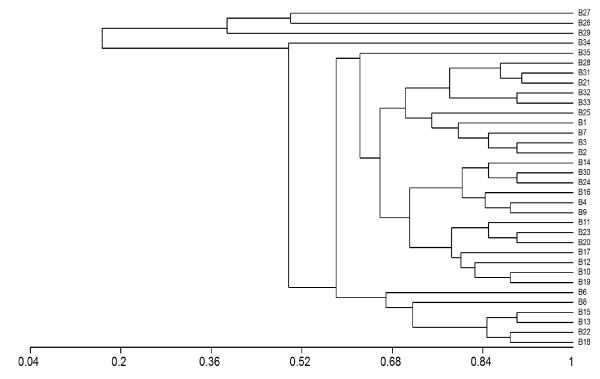
**Cluster analysis of API 20NE results**. B: Biotype 1 to 35- numbers assigned to API 20NE profile, isolates belonging to each biotype can be seen in Table 1. Scale is a measure of the phenotypic relatedness of isolates.

A cluster analysis was carried out using the API 20 NE results and can be seen in Figure 1. The results indicated that the isolates studied are phenotypically very different (The list of tests in the API 20NE can be seen in Additional File 1 Table S1). The 35 biotypes identified are very different with similarity between some of the biotypes being as low as 0.2. The 35 biotypes did not break down based on environment of isolation. These results contradict the results of both the Remel RapID NF Plus and the Vitek NFC, which indicated that *R. pickettii *was a phenotypically homogenous species with the same phenotypic pattern being found in most isolates. All results are presented in Table 3 and Additional File 1, Table S1. A Simpson's diversity of 0.9813 was calculated for this study using the API 20NE results [30].

### Genotypic characterisation

**Figure 2:**
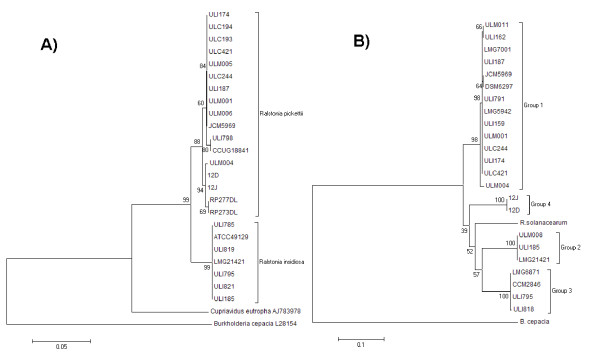
**Phylogenetic trees**. A) Phylogenetic tree of *R. pickettii *and *R*. *insidiosa *16*S*-23*S *ISR of nineteen sequenced isolates and sequence data available on the Genbank database. The tree was rooted with the ISR of *Ralstonia solanacearum *(Genbank Accession No AJ277280), *Cupriavidus necator *(AJ783978) and *Burkholderia cepacia *(L28154). B) Phylogenetic tree of *R. pickettii *and *R*. *insidiosa fliC *genes of nineteen sequenced isolates and sequence data available on the Genbank database. The tree was rooted with the *fliC *of *Burkholderia cepacia *(L28154). Cluster analysis was based upon the neighbour-joining method. Numbers at branch-points are percentages of 1000 bootstrap resamplings that support the topology of the tree.

Four different DNA-based typing methods (ISR and *fliC *gene sequencing, RAPD-PCR and BOX-PCR) were used to compare the isolates at a molecular level. With the analysis of the 16S-23S rDNA ISR a PCR product of approximately 860 bp was obtained for all isolates indicating that the spacer region is highly similar in length in all isolates (data not shown). Sequencing of the ISR of 19 isolates identified phenotypically as *R. pickettii*, and the type strain of *R. insidiosa *was carried out. The sequence of several isolates indicated that these were more closely related to *R. insidiosa *than to *R. pickettii *sharing greater homology with the *R. insidiosa *type strain confirming the results obtained from the species-specific PCR reaction (Figure 2a). The ISR comprised a length of 513bp for *R. pickettii *and 515bp for *R. insidiosa*. The sequence similarity of the *R. pickettii *isolates compared to the *R. pickettii *type strain LMG5942 ranged from 98-100% (Figure 2a) and for all *R. insidiosa *isolates it was 95% (Figure 2a). All ISR sequences had a GC content of ~52.5%. The *Ralstonia *ISR spacer region contains two tRNA genes: tRNA^Ile ^and tRNA^Ala ^comprising 77 and 78 bp respectively. This is a common feature of the ISR in *rrn *operons in Gram-negative bacteria [45] including *R. pickettii *[46]. The order observed for sequences generated from our *Ralstonia *isolates was 16*S *rRNA - tRNA^Ile ^- tRNA^Ala ^-23*S *rRNA. The nucleotide sequences of tRNA^Ile ^were identical in all isolates and the tRNA^Ala ^gene differed by one nucleotide between *R. pickettii *and *R. insidiosa *in the isolates studied. The phylogenetic tree analysis in Figure 2a, supports the positioning of *R. pickettii *and *R. insidiosa *as two separate groups (bootstrap values of 91%), with *B. cepacia *as an out-group. The isolates identified as *R. pickettii *themselves divide into two different groups (bootstrap value of 99%). However the division into groups did not correlate to clinical or environmental association or indeed on their isolation location.

Sequencing was carried out on the *fliC *gene of sixteen randomly selected isolates of *R. pickettii*, and the type strain of *R. insidiosa*. The phylogenetic analysis of the *fliC *gene can be seen in Figure 2b, with the isolates divided into two branches with *B. cepacia *as an out-group. The isolates identified as *R. insidiosa *in-group two grouped together with groups three and four. These however were not supported by high bootstrapping values. Group 1 is made up of *R. pickettii *isolates from clinical and environmental sources with 97-100% similarity to the *R. pickettii *type strain. Group 2 is made up of *R. insidiosa *with 85% similarity to the *R. pickettii *type strain*; *Group 3 is made up of both *R. insidiosa *and *R. pickettii *with 86-87% similarity to the *R. pickettii *type strain and Group 4 is made up of the available sequenced *R. pickettii *strains with 87% similarity to the *R. pickettii *type strain. The division of the groups did not correlate to clinical or environmental association or on their isolation location. These results indicate that there is variation in the flagellin gene of *R. pickettii*.

### RAPD PCR results and analysis

RAPD analysis was carried out using four different primers, three of which (P3, P15 and M13) have been shown to discriminate between closely related strains of *Ralstonia *spp. including *R*. *mannitolilytica *and *Cupriavidus pauculus *[*Ralstonia paucula*] [47,48]. The reproducibility of the RAPD method was tested by repeating the RAPD assays at least three times for each primer used (data not shown). The results revealed that apart from some variations in the band intensity, no significant differences were observed between the profiles obtained, confirming the reproducibility of the method.

**Figure 3:**
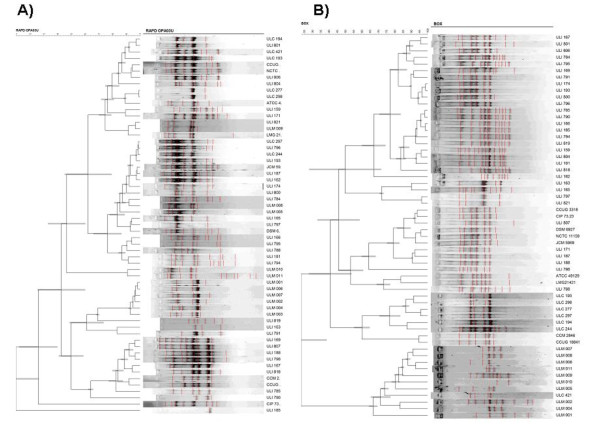
**RAPD analysis with primer OPA03U and BOX analysis**. A) RAPD analysis with primer OPA03U B) BOX analysis. Dendrogram of fifty-nine isolates of *R. pickettii *and *R*. *insidiosa *by the Pearson correlation using the UPGMA linkage method.

Fifty-nine isolates of *R*. *pickettii *and *R. insidiosa *were characterised by RAPD analysis using all four primers and all isolates were placed into genotypes (Table 3). Percent similarities based on the Pearson correlation coefficients and clustering by the UPGMA method for these isolates using the OPA03U primer is presented in Figure 3a. Dendograms for the other primers (P3, P15 and M13) are presented in Additional File 2, Figure S1, S2 and S3. Fragments ranged from approximately 100 to 1800 bp for all primers. Clusters were distinguished at a similarity cut-off level of 80%. No major differentiation between the clinical, industrial, laboratory purified water and type strains could be observed, as these all fell into separate groups (Table 3) with each primer. For each of the primers there were a number of groups, with M13 there were twenty-one groups, OPA3OU there were 15 groups, P3 there were twenty-five groups and with primer P15 there were twenty-one groups. The clinical isolates grouped together with two of the primers, with M13 they clustered together in Genotype A with the type strain JCM5969, three laboratory water isolates and three industrial water isolates, with P3 they clustered together in Genotype × with nine of the industrial water isolates (including the industrial isolates that grouped together with the clinical isolates in Genotype A with the M13 primer), with primers P15 and OPA30U they fell into several clusters six with P15. The industrial purified water isolates also fell into different groups with all four primers. There were nine groups with primer P15, thirteen groups with primer M13, fifteen groups with primer P3 and eleven groups with primer OPA3OU. The laboratory purified water isolates fell into two different groups with primer P15, six groups with primer M13, five groups with primer P3 and three groups with primer OPA3OU. The isolates identified as *R. insidiosa *failed to group together with any of the RAPD primers. With the P15 primer there is one large group that contained all the type strains, the soil strains, ten of laboratory water purified isolates and the industrial water isolates, no other primer produced such a large group. The diversity of the bacterial populations studied was calculated using Simpson's Index of Diversity (Di) [30] and the results of each individual primer were M13-0.897, OPA3OU-0.899, P3-0.918 and P15-0.771. The average diversity for the four primers was 0.869. An index (**D**) of 0.90 or greater is a desirable property of a typing scheme [30]. As can be seen from the results only primer P3 with a **D **of 0.918 produced a significant D index. The D value indicates that primer P3 would be the best primer to carry out further studies into the diversity of *R. pickettii *in the future as it is the most discriminatory primer of the four tested.

### BOX-PCR results and analysis

The fifty-nine isolates of *R*. *pickettii *and *Ralstonia insidiosa *were characterised by the BOX-PCR analysis using the BOX-A1R primer [39]. Repeatability of the BOX-PCR was considered good as the isolates showed identical profiles in three independent experiments (data not shown). The results revealed that while there were some variations in the band intensities, no significant differences were observed between the profiles obtained. Percent similarities based on the Pearson correlation coefficients and clustering by the UPGMA method for these isolates are presented in Figure 3b. Clusters were distinguished at a similarity cut-off level of 80%. With the BOX primer eighteen groups were found at this cut-off level. Fragments ranged from approximately 300 to 3000 bp for all primers. The number of groups can be seen in Table 4. The groups, in contrast to the RAPD primers, mostly contained bacteria isolated from the same environments e.g. Group F clustered all the type strains together, Group M the soil strains and groups K, L and P the clinical isolates. The industrial isolates grouped together in-group A, B, C, D, E, G and J. The laboratory water isolates grouped together in groups N, O, Q and R. As with all four RAPD primers the isolates identified as *R. insidiosa *failed to group together. The Di using BOX-A1R was 0.915. These various primers and techniques demonstrated the limited diversity of the *R*. *pickettii*.

**Table 4:** No.of Groupings with Four Different RAPD Primers and Box Primer

Primer	No. of Groupings	Discrimination index
M13	21	0.897
OPA3OU	15	0.899
P3	25	0.918
P15	21	0.771
BOX	18	0.915

## Discussion

In the course of this study a number of bacteria previously identified phenotypically as *R. pickettii *were subsequently identified as *R. insidiosa *using species-specific PCR. These bacteria are hard to distinguish from each other phenotypically [49]. *R. insidiosa*, the closest related bacteria to *R. pickettii *[33], has been isolated from the respiratory tracts of cystic fibrosis patients [33], river and pond water, soil, activated sludge [33] and has also been detected in water distribution systems [50] and laboratory purified water systems [3]. It has also been the causative agent of two cases of serious hospital infection in two immunocompromised individuals [51].

Each of the four DNA-based fingerprinting and sequencing methods were suitable for distinguishing and grouping the isolates, although the sensitivity of the methods varied. Of the three phenotypic methods examined, the API 20NE system was more discriminatory than the Remel RapID NF Plus system or the Vitek NFC. However, the Remel RapID NF Plus system and the Vitek NFC did prove more useful for the accurate identification of *R. pickettii *isolates, as previously reported [52]. The API 20NE gave thirty-five different biotypes for fifty-nine isolates (Table 3, Figure 1), which grouped together isolates from different environments. These results broadly agree with those of Dimech *et al *who found homogeneity in physiological parameters [25].

Genotypic studies carried out by both Dimech *et al*. and Chetoui *et al*. hinted that *R. pickettii *also had genotypic homogeneity [25,26]. This was investigated in this study using the methods described above. Our data based on the sequence of 16*S*-23*S *spacer regions of nineteen isolates indicated that *Ralstonia pickettii *is a homogenous species with little difference between isolates from different environmental niches. Clearly using these methods we can however determine differences between *R. pickettii *and *R. insidiosa*.

The *fliC *gene has been used for bacterial strain differentiation in multiple studies such as for *Ralstonia solanacearum *[35] and *Burkholderia cepacia *complex [53]. Four different types of flagellin gene have been found in *R. pickettii *isolates analysed in this study (Groups 1, 2, 3 and 4). This is similar to data from *P. aeruginosa *where two different types of *fliC *gene have been found [54] and from the *B. cepacia *complex where again two different types of *fliC *gene have also been found [55,56]. The *fliC *gene appears however not to be useful for distinguishing between *R. pickettii *and *R. insidiosa *based on our findings. The division of the groups did not correlate to clinical or environmental association or to their location of isolation. The reasons for the variation between the 16*S*-23*S *spacer region and the *fliC *gene could be potentially due to the structure of the *fliC *gene. This is demonstrated by *Burkholderia *flagellin sequences, which exhibit high levels of homology in the conserved terminal regions but differ considerably in the central region [57]. Variation is a common feature of flagellin proteins, which are believed to fold into a hairpin-like conformation, with the terminal domains being responsible for defining the basic filament structure lying on the inner surface and the central, variable region being surface exposed [58].

In a previous epidemiological study involving sixteen isolates of *R. pickettii*, eight different RAPD profiles were observed for isolates coming from blood culture, distilled water and an aqueous chlorhexidine solution [16]. In another study, involving fourteen isolates of *R. pickettii *from various biological samples the same RAPD pattern was found in all instances [59], while Pasticci *et al.*, carried out a study involving fifteen isolates of *R. pickettii *that gave three patterns [27]. The results of our study with a larger number of isolates indicated that there is some diversity in the studied populations but that this is limited and isolates from different environments grouped together.

The results obtained with BOX-PCR showed nineteen different profiles among the fifty-nine isolates examined again demonstrating limited diversity (Figure 3b). To the best of our knowledge this is the first reported study of the diversity of *R. pickettii *and *R. insidiosa *carried out with BOX-PCR. A similar study carried by Coenye *et al.*, on ninety-seven *B. cepacia *Genomovar III isolates found 20 different patterns with a DI value of 0.821 [60].

The molecular fingerprinting methods used here yielded rapid and reproducible fingerprints for clinical and environmental isolates of *R. pickettii *and *R. insidiosa*. Presently, little is known regarding the source of *R. pickettii *isolates occurring in hospital environments. Investigations by other authors have reported no evidence of patient-to-patient transmission, and they suggest that multiple independent acquisitions from environmental sources could be an important mode of transmission of *R. pickettii *[5]. The most common sites of contamination were blood-sampling tubes, dialysis machines, nebulizers and other items frequently in contact with water [5].

## Conclusions

BOX-PCR and RAPD typing was found to be more discriminatory than the typing of genes in *R. pickettii *such as the *fliC *gene or the ISR. The majority of isolates were shown to possess similar genotypes by both BOX and RAPD-PCR (Figure 3a, b). The limited diversity of *R. pickettii *and *R. insidiosa *isolates observed in this study for fifty-nine isolates is consistent with previous findings and indicates that *R. pickettii *appears to be a genotypically and phenotypically homogeneous species.

## Authors' contributions

MPR conceived the study and its design, carried out the experimental work, performed the analysis and interpretation of the data and wrote the manuscript. JTP participated in conceiving the study and in its design and participated in writing the manuscript. CAA participated in conceiving the study, its design, and participated in writing the manuscript. All authors read and approved the final manuscript. The authors declare no conflict of interest.

## Supplementary Material

Additional file 1 **Table S1**. API 20NE and Remel Rapid NF Plus Codes for isolates used in this study and identifiers for biochemical tests. Click here for file

Additional file 2 **Figure S1, S2, S3**. Dendograms for primers M13, P3 and P15 that were not included in the paper. Click here for file
