# Supplementary material for: Genotypic and phenotypic diversity of Ralstonia pickettii and Ralstonia insidiosa isolates from clinical and environmental sources including High-purity Water. Diversity in Ralstonia pickettii
Source: BMC Microbiol. 2011 Aug 30;11:194. doi: 10.1186/1471-2180-11-194 (PMC3175462; doi:10.1186/1471-2180-11-194)
Supplement: Additional file 1 — Table S1. API 20NE and Remel Rapid NF Plus Codes for isolates used in this study and identifiers for biochemical tests. [file 1471-2180-11-194-S1.DOC]

Table S1: API 20NE and Remel Rapid NF Plus Codes

| **Strain** | API 20NE Code | % ID *R. pickettii* | RapID NF Plus Code | % ID *R. pickettii* | % ID *R. pickettii* |
| --- | --- | --- | --- | --- | --- |
| ***Ralstonia pickettii*** | | | | | |
| JCM5969 | 1041465 | 99.00 | 400414 | 99.94 | *R. pickettii* |
| NCTC11149 | 1041455 | 95.10 | 400414 | 99.94 | *R. pickettii* |
| DSM6297 | 1041455 | 95.10 | 400414 | 99.94 | *R. pickettii* |
| CCUG3318 | 1041555 | 91.10 | 400414 | 99.94 | *R. pickettii* |
| CIP73.23 | 1041555 | 91.10 | 400414 | 99.94 | *R. pickettii* |
| CCUG18841 | 1055555 | 00.00 | 400616 | 99.71 | *R. pickettii* |
| CCM2846 | 1055555 | 00.00 | 400616 | 99.71 | *R. pickettii* |
| ULI187 | 1041565 | 97.70 | 404614 | 98.34 | *R. pickettii* |
| ULI188 | 1041455 | 95.10 | 404414 | 99.99 | *R. pickettii* |
| ULI798 | 0045445 | 95.10 | 404414 | 99.99 | *R. pickettii* |
| ULI807 | 0045455 | 84.10 | 404414 | 99.99 | *R. pickettii* |
| ULI171 | 0045455 | 84.10 | 404414 | 99.99 | *R. pickettii* |
| ULI788 | 0245455 | 80.40 | 400414 | 99.94 | *R. pickettii* |
| ULI800 | 0245455 | 80.40 | 404414 | 99.99 | *R. pickettii* |
| ULI169 | 0245455 | 80.40 | 404414 | 99.99 | *R. pickettii* |
| ULI165 | 1045455 | 67.90 | 404414 | 99.99 | *R. pickettii* |
| ULI174 | 1045455 | 67.90 | 404614 | 98.34 | *R. pickettii* |
| ULI193 | 0050577 | 61.70 | 400614 | 98.38 | *R. pickettii* |
| ULI796 | 1241455 | 60.00 | 404614 | 98.34 | *R. pickettii* |
| ULI801 | 0044455 | 56.90 | 404414 | 99.99 | *R. pickettii* |
| ULI791 | 0044455 | 56.90 | 404414 | 99.99 | *R. pickettii* |
| ULI790 | 0255455 | 44.80 | 404614 | 98.34 | *R. pickettii* |
| ULI818 | 1045555 | 39.50 | 400414 | 99.94 | *R. pickettii* |
| ULI804 | 0055455 | 24.50 | 404614 | 98.34 | *R. pickettii* |
| ULI159 | 1200004 | 00.00 | 400414 | 99.94 | *R. pickettii* |
| ULI806 | 1044444 | 00.00 | 404414 | 99.99 | *R. pickettii* |
| ULI167 | 1050555 | 00.00 | 400414 | 99.94 | *R. pickettii* |
| ULI162 | 1145455 | 00.00 | 404414 | 99.99 | *R. pickettii* |
| ULC298 | 0051574 | 90.10 | 404414 | 99.99 | *R. pickettii* |
| ULC297 | 0050557 | 70.03 | 400414 | 99.94 | *R. pickettii* |
| ULC277 | 0050577 | 61.70 | 404414 | 99.99 | *R. pickettii* |
| ULC244 | 0050555 | 56.70 | 400414 | 99.94 | *R. pickettii* |
| ULC193 | 0050555 | 56.70 | 404614 | 98.34 | *R. pickettii* |
| ULC194 | 0050555 | 56.70 | 404414 | 99.99 | *R. pickettii* |
| ULC421 | 0050575 | 28.50 | 404414 | 99.99 | *R. pickettii* |
| ULM001 | 1041455 | 95.10 | 404416 | 99.99 | *R. pickettii* |
| ULM002 | 1041455 | 95.10 | 400416 | 99.99 | *R. pickettii* |
| ULM003 | 1041457 | 88.60 | 400406 | 99.28 | *R. pickettii* |
| ULM004 | 1041555 | 91.10 | 400416 | 99.99 | *R. pickettii* |
| ULM005 | 1040455 | 95.10 | 600416 | 00.00 | *R. pickettii* |
| ULM006 | 1041455 | 95.10 | 400406 | 99.28 | *R. pickettii* |
| ULM007 | 1041455 | 95.10 | 400416 | 99.99 | *R. pickettii* |
| ULM010 | 1041575 | 99.40 | 400416 | 99.99 | *R. pickettii* |
| ULM011 | 1041575 | 99.40 | 400416 | 99.99 | *R. pickettii* |
| ***Ralstonia insidiosa*** | | | | | |
| LMG21421 | 0050577 | 61.70 | 400414 | 99.94 | *R. insidiosa* |
| ATCC49129 | 0040475 | 92.40 | 404414 | 99.99 | *R. insidiosa* |
| ULI821 | 0045455 | 84.10 | 400414 | 99.94 | *R. insidiosa* |
| ULI797 | 0045455 | 84.10 | 404614 | 98.34 | *R. insidiosa* |
| ULI785 | 0045457 | 53.10 | 404414 | 99.99 | *R. insidiosa* |
| ULI181 | 1045555 | 39.50 | 404414 | 99.99 | *R. insidiosa* |
| ULI794 | 1141455 | 06.40 | 400404 | 34.18 | *R. insidiosa* |
| ULI185 | 1251575 | 05.70 | 404614 | 98.34 | *R. insidiosa* |
| ULI166 | 1054555 | 00.00 | 400414 | 99.94 | *R. insidiosa* |
| ULI819 | 1372004 | 00.00 | 404414 | 99.99 | *R. insidiosa* |
| ULI784 | 1310000 | 00.00 | 404414 | 99.99 | *R. insidiosa* |
| ULI163 | 1245555 | 00.00 | 404614 | 98.34 | *R. insidiosa* |
| ULI795 | 1041645 | 00.00 | 404614 | 98.34 | *R. insidiosa* |
| ULM008 | 0041455 | 80.20 | 400416 | 99.99 | *R. insidiosa* |
| ULM009 | 0041455 | 80.20 | 400416 | 99.99 | *R. insidiosa* |

**Figure 1:** Results sheet for the Remel Rapid NF Plus

**Table 1:** Biochemical tests for the Remel Rapid NF Plus

| **Test** | **Reaction/Enzymes** |
| --- | --- |
| ADH | Arginine dihydrolase |
| TRD | Thiosulfate utilization |
| EST | Fatty acid esterase production |
| PHS | Hydrolysis of *p*-nitrophenylphosphoester |
| NAG | Hydrolysis of *p*-nitrophenyl-*N*-acetyl-β,D-glucosaminide |
| α-Glu | Hydrolysis of *p*-nitrophenyl-α,D-glucoside |
| β-Glu | Hydrolysis of *p*-nitrophenyl-β,D-glucoside |
| ONPG | Hydrolysis of *o*-nitrophenyl-β,D-galactoside (ONPG) |
| URE | Urease production |
| GLU | Glucose fermentation |
| PRO | Hydrolysis of proline-β-naphthylamide |
| PYR | Hydrolysis of pyrrolidine-β-naphthylamide |
| GGT | Hydrolysis of γ-glutamyl-β-naphthylamide |
| TRY | Hydrolysis of tryptophane-β-naphthylamide |
| BANA | Hydrolysis of *N-*benzyl-arginine-β-naphthylamide |
| IND | Indole production |
| NO_3_ | Nitrate production |
| OXI | Cytochrome oxidase |

**Figure 2:** Results sheet for the API 20NE


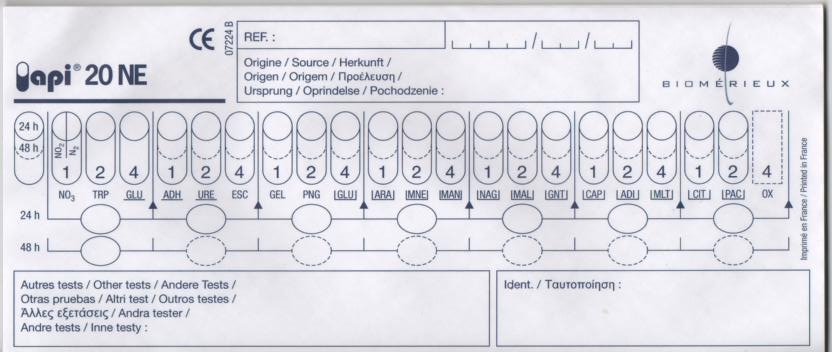


**Table 2:** Biochemical tests for the API 20NE
