# Supplementary material for: Genotypic and phenotypic diversity of Ralstonia pickettii and Ralstonia insidiosa isolates from clinical and environmental sources including High-purity Water. Diversity in Ralstonia pickettii
Source: BMC Microbiol. 2011 Aug 30;11:194. doi: 10.1186/1471-2180-11-194 (PMC3175462; doi:10.1186/1471-2180-11-194)
Supplement: Additional file 2 — Figure S1, S2, S3. Dendograms for primers M13, P3 and P15 that were not included in the paper. [file 1471-2180-11-194-S2.DOC]

**Fig S1:** RAPD primer M13. Dendrogram of fifty-nine strains of *R. pickettii* and *R. insidiosa* by the Pearson correlation using the UPGMA linkage method

**Fig S2:** RAPD primer P3. Dendrogram of fifty-nine strains of *R. pickettii* and *R. insidiosa* by the Pearson correlation using the UPGMA linkage method

**Fig S3:** RAPD primer P15. Dendrogram of fifty-nine strains of *R. pickettii* and *R. insidiosa* by the Pearson correlation using the UPGMA linkage method
